# Superior Strength and Multiple Strengthening Mechanisms in Nanocrystalline TWIP Steel

**DOI:** 10.1038/s41598-018-29632-y

**Published:** 2018-07-25

**Authors:** Jung Gi Kim, Nariman A. Enikeev, Jae Bok Seol, Marina M. Abramova, Marina V. Karavaeva, Ruslan Z. Valiev, Chan Gyung Park, Hyoung Seop Kim

**Affiliations:** 1 Department of Materials Science and Engineering, Pohang University of Science and Technology (POSTECH), Pohang, 37673 South Korea; 2 Institute of Physics of Advanced Materials, Ufa State Aviation Technical University, Ufa, 450000 Russia; 3 Saint Petersburg State University, St. Petersburg, 198504 Russia; 4 National Institute of Nanomaterials Technology (NINT), POSTECH, Pohang, 37673 South Korea; 5 Center for High Entropy Alloys, POSTECH, Pohang, 37673 South Korea

## Abstract

The strengthening mechanism of the metallic material is related to the hindrance of the dislocation motion, and it is possible to achieve superior strength by maximizing these obstacles. In this study, the multiple strengthening mechanism-based nanostructured steel with high density of defects was fabricated using high-pressure torsion at room and elevated temperatures. By combining multiple strengthening mechanisms, we enhanced the strength of Fe-15 Mn-0.6C-1.5 Al steel to 2.6 GPa. We have found that solute segregation at grain boundaries achieves nanograined and nanotwinned structures with higher strength than the segregation-free counterparts. The importance of the use of multiple deformation mechanism suggests the development of a wide range of strong nanotwinned and nanostructured materials via severe plastic deformation process.

## Introduction

Strong metals have been exploited indispensably in the manufacturing of tools for architecture, military, and transportation vehicles. Since the high strength is important to sustain under the extreme conditions with a high reliability, various kinds of high strength materials were applied to the specific fields. For example, ultrafine grained titanium alloys were developed to enhance the reliability of permanent implants^1^, and the nanosteels were applied to manufacture the micro bolts^2^. These examples represent that there is a demand for high strength materials, and the development of the strongest material in the bulk state is one of the challengeable issues. For years, the highest strength of commercial engineering metallic alloys (often achieved at the expense of introducing costlier elements^3^) has been screened down to values of 2.0 GPa^4^, exceeded only in cases of specific alloys such as pearlitic wires and high carbon martensitic steels^5,6^.

Material strength is determined by the movability of the internal lattice dislocations, which are linear irregularities in the crystal lattice, during plastic deformation^7–9^, i.e., impeding lattice dislocation motion leads to an increase in the material strength and superior strength can be provided by the maximal amount of obstacles. Among the material strengthening mechanisms, a regular or very dense array of deformation twins is a successful strategy to manipulate the internal defect landscape of nanometer-scale materials to improve the strength without ductility reduction^10–12^. This strategy is essential for the face-centered cubic (fcc; austenite) structure in which a high density of coherent twin boundaries act as conventional coherent grain boundaries (GBs) in strengthening materials by impeding lattice dislocation motion and thereby providing strength to materials^8,9^. Based on this twin boundary-dislocation interaction, the strength of twinned materials increases as the twin thickness decreases^10^.

An alternative route towards a substructure architecture consisting of nanosized grains is to exploit GB segregation engineering with severe plastic deformation^13,14^ or via heat treatment^15^. Structural defects such as GBs or dislocations are prone to attract sufficient solute segregation by means of high transport coefficients for diffusing atoms, enabling changes in energy, structure, and cohesion, and even promoting local solid phase transformation^9,16^. In particular, trace element concentration (especially carbon; C) segregated at the grain boundary in many Fe based alloys increases the Hall-Petch coefficient^17^. Based on the dislocation pile-up model, GB segregation of solute atoms increases the emission stress for the mobile dislocations^18^.

The multiple strengthening mechanisms outlined here are realized by severe plastic deformation (SPD) of high-pressure torsion (HPT)^19^. Since HPT is based on high torsional shearing strain combined with high hydrostatic pressure, a coarse-grained polycrystalline material is transformed into a nanocrystalline (NC) counterpart with the same chemical composition. Hence, the HPT-treated NC materials can contain high densities of dislocations, coherent nanotwins, and coherent grain boundaries per unit volume. In general, HPT is considered as the most effective model method to produce nanostructures in order to reveal the hardening mechanisms in nanocrystalline steels. Due to the small dimensions of the produced samples, the conventional HPT specimens could not be used in a practical application for material fabrication. However, recent HPT studies represent upscaled or modified techniques to produce large-scale disks and cone-shaped specimen^20,21^ as well as strips or rods^22–24^. Such a development allows HPT to be considered as a plastic forming method for manufacturing the parts of nanocrystalline materials.

Moreover, applying HPT at elevated temperature (573 K; HT) may be more efficient in terms of trace element segregation to the structural defects as compared to the room-temperature (RT) HPT^25^. This effect enables grain refinement, nanotwinning, and segregation engineering in the NC solid solutions, particularly in an fcc Fe-based alloy. On the other hand, during the same material processing subject to RT-HPT, superimposed nanotwins on the NC structure results into the formation of either body-centered cubic (bcc; α′-martensite) or hexagonal close-packed (hcp; ε-martensite) crystals via local solid phase transformation, but no solute segregation. Taking a step in this direction, we have performed transmission electron microscopy (TEM) combined with atom probe tomography (APT) to resolves solute segregation with near-atomic spatial resolution and high detection sensitivity in 3D^9,18,26^.

## Results

### Mechanical testing

**Figure 1:**
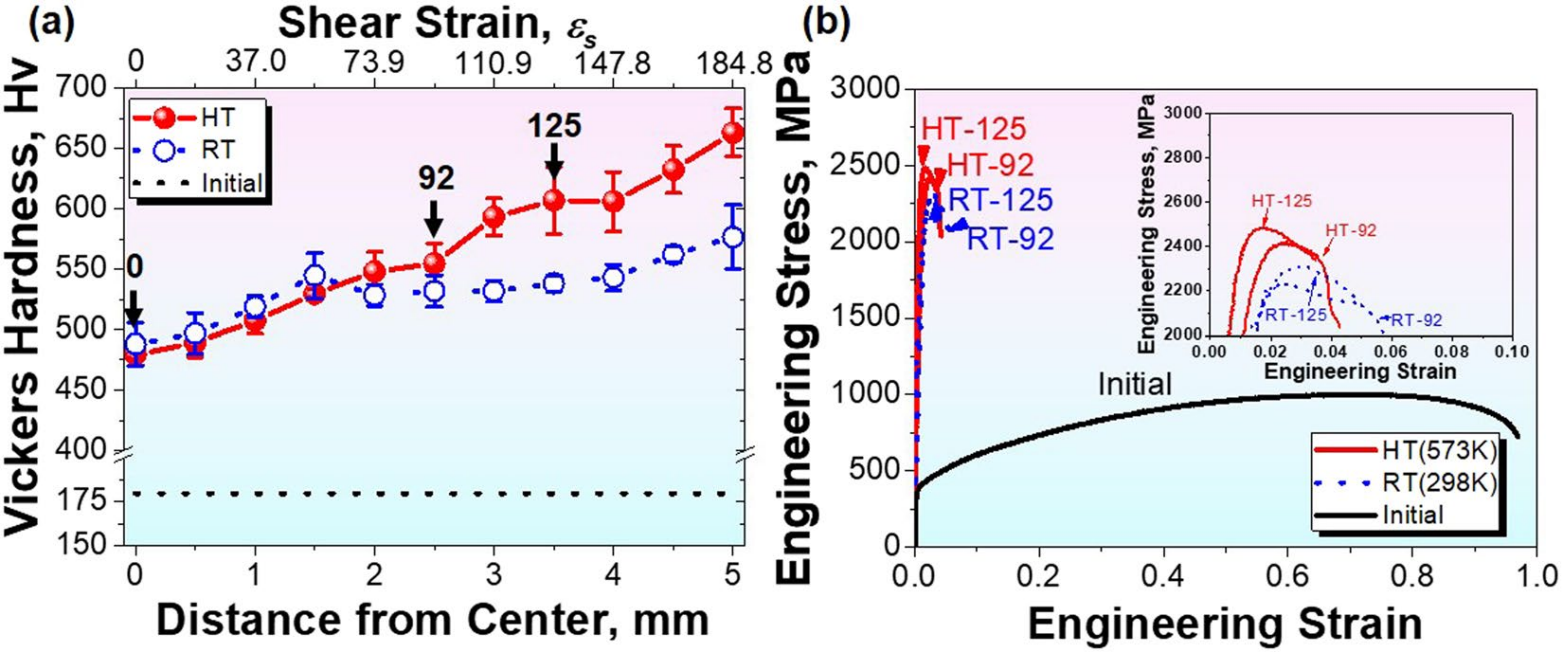
Mechanical properties of the HPT-processed samples. **(a)** Microhardness distributions as a function of *ε*_*s*_, i.e. distance from the center. Hardness evolution of the HPT-process samples is classified: for *ε*_*s*_ < 92, the sample strengths of HT- (open circles) and RT-HPT (closed circles) increase with increasing *ε*_*s*_; for 92 ≤ *ε*_*s*_ ≤ 125, a strength difference evolves; for *ε*_*s*_ = 125, a significant strength enhancement occurs in HT-HPT. **(b)** The stress-strain curves of the RT-92, RT-125, HT-92, and HT-125 samples.

Stepwise strengthening mechanisms manipulate the mechanical properties of both the RT-HPT and HT-HPT samples. Figure 1(a,b) represent the hardness and tensile properties of an HT-HPT and RT-HPT sample material with respect to the amount of shear strain. The amount of applied shear strain was calculated as a function of distance from the center (*D*_*f-c*_) of the materials treated by RT- and HT-HPT. Two distinct features regarding the hardness dependence of the two samples with respect to applied shear strain (hereafter referred to as *ε*_*s*_) are observed^27^. The first is that the hardness of the HPT-treated samples produced here increases with increasing *ε*_*s*_. This hardness increment is owing to grain size refinement via SPD, i.e., an increase in the *D*_*f-c*_ enables high *ε*_*s*_, leading to a decrease in average grain size. The second feature is that the difference in the *ε*_*s*_ dependence of the hardness between the two samples becomes noticeable at *ε*_*s*_ ≥ 92 (*D*_*f-c*_ ≥ 2.5 mm). In the *ε*_*s*_ range of 92 to 125, the hardness of an HT-HPT material (red circles) increases to approx. 600 ± 50 Hv, while the values < 550 Hv remain for an RT-HPT sample (blue open circles). Although the extra hardness increment occurs at *ε*_*s*_ ≥ 140, this enhancement is originated from the geometrical effect of the HPT-treated disk samples^28^. To exclude this geometrical effect, in this research, only the *ε*_*s*_ < 140 region is considered.

In addition to the hardness distribution, we demonstrated the significant effects of both RT- and HT-HPT treatments on the uniaxial tensile stress-strain curves of the NC materials with respect to applied shear strains, in particular for *ε*_*s*_ values of 92 and 125. For simplicity, each RT- or HT-HPT sample is identified by its shear strain; for instance, the RT-HPT-treated sample at *ε*_*s*_ = 92 is referred to as RT-92. The mechanical properties are characterized by means of ultimate tensile strength (TS) and elongation. As *ε*_*s*_ increased from 92 to 125, the TS increased from 2.25 GPa for an RT-92 material to 2.3 GPa for an RT-125 sample, while reaching values of 2.6 GPa for the HT-125 sample. The elongation of all HPT samples remained in the 4−6% range (2–3% uniform elongation; see the Supplementary Fig. 1), irrespective of *D*_*f-c*_ or the amount of *ε*_*s*_ caused by the SPD process. Although the elongation of HPT samples is incorrect due to the small tensile specimen, this result shows that the HPT samples maintain plasticity.

### TEM observation

**Figure 2:**
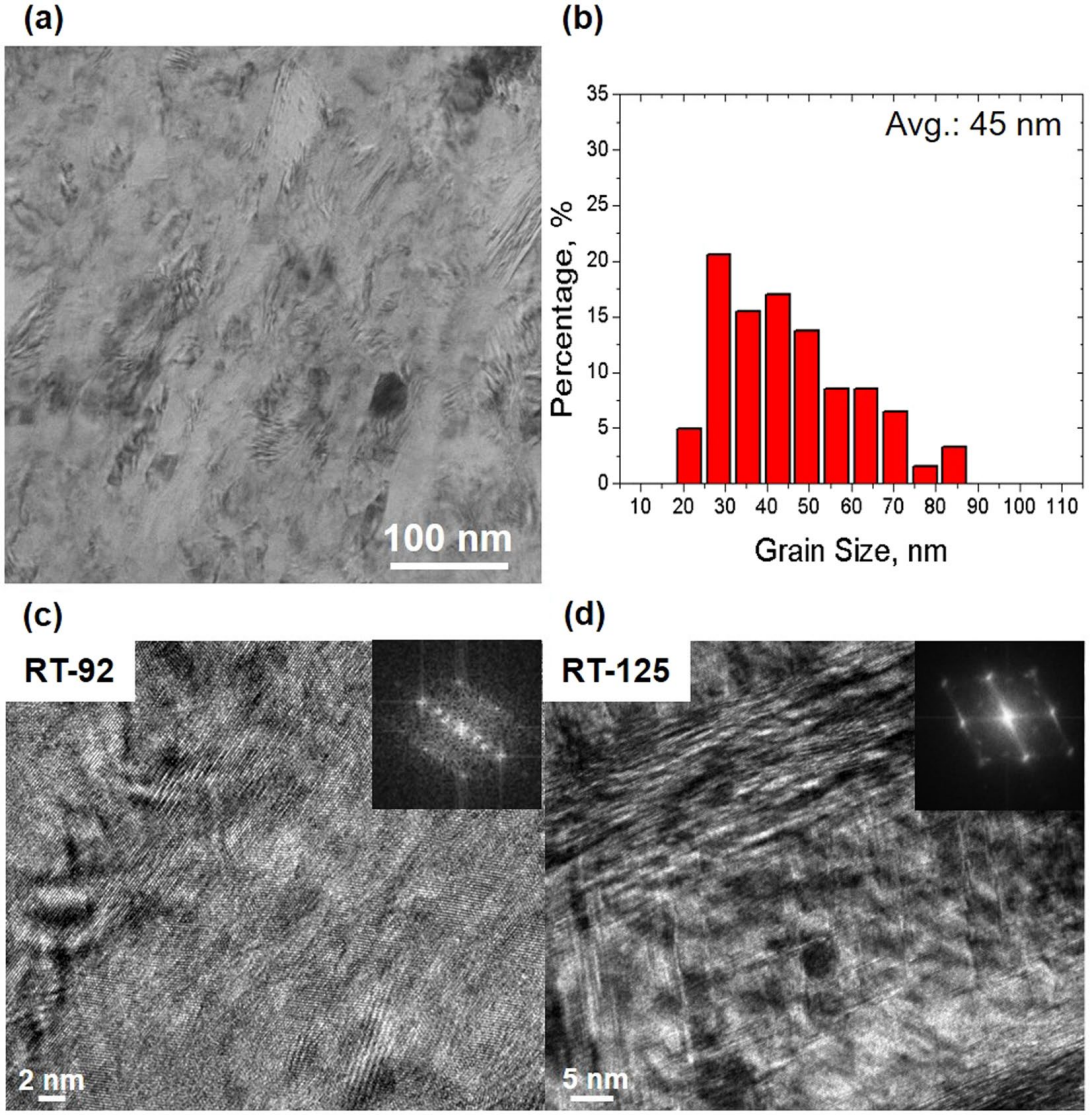
RT-HPT microstructures showing solid phase transformation with respect to applied shear strains. **(a)** Low-magnification bright-field micrograph of RT-HPT samples. **(b)** Grain size distribution (average grain size 45 nm) determined from HRTEM images. **(c)** HRTEM images and corresponding selected area electron diffraction patterns with [011]_fcc_ zone axis shown as inset for RT-92 samples. Solid phase transformation occurs from γ to ε-martensite. **(d)** The HRTEM micrograph for the RT-125 samples shows phase transformation from γ to ε- and ε- to α′-martensite at the intersection point of two ε-crystals (for detailed analysis, see the Supplementary Fig. 2).

The bright-field TEM micrographs of the RT-HPT samples deformed at the *ε*_*s*_ values of 92 and 125, and corresponding electron diffraction patterns are shown in Fig. 2. At *ε*_*s*_ = 92, we observed equiaxed fcc host grains with an average size of 45 nm. In addition, hcp ε-martensite product crystals were observed in the local regions of the host matrix. The electron diffraction pattern indexed as the [011]_fcc_ crystal, obtained from the targeted area, shows the diffraction reflections related to the ε-crystal that obeys the orientation relationship with the adjacent fcc phase. This indicates that during RT-HPT at *ε*_*s*_ = 92, the fcc host solid phase transforms locally to the hard solid phase of ε-martensite, i.e., fcc austenite (γ) → ε-martensite^29^. Since the ε-martensite is generated by the super-position of stacking faults in the γ-austenite^30,31^, the strain-induced ε-martensite contains high dislocation density and achieves higher strength than γ-austenite. As a result, the existence of solid phase transformation of γ → ε-martensite provides additional strength to RT-92 by the transformation-induced plasticity (TRIP) effect^32^.

The driving force for the formation of ε-martensite in the fcc host matrix of RT-HPT originates from the high excess deformation energy of large shear strains under a hydrostatic pressure, and it becomes sufficiently large that the ε-phase nucleates and grows easily at RT. Though the high Mn steels have high γ stability, Adachi *et al*. have observed the γ → ε-martensite transformation during HPT^33^. The TEM image of the RT-125 materials shown in Fig. 2(d) indicates the presence of multiple phases, such as fcc host γ, hcp product ε-martensite, and bcc product α′-martensite. The reflection spots corresponding to the bcc crystal structure are visible in the [011]_fcc_ diffraction pattern (Details of the orientation relationship between the products and the host phase are provided in Supplementary Fig. 2). In the *ε*_*s*_ = 125, an increase in the transformation frequency of γ → ε leads to a large density of intersecting zones of ε-phase crystals. These zones are known to be effective as conventional nucleation sites that enable the formation of bcc α′-martensite at the expense of ε-martensite (ε → α′)^34^. Considering the *ε*_*s*_ dependence of the mechanical behavior in RT-HPT, it is recognized that the ε → α′ transformation in the RT-125 sample may lead to relative softening behavior compared to the γ → ε transformation in the RT-92 material.

**Figure 3:**
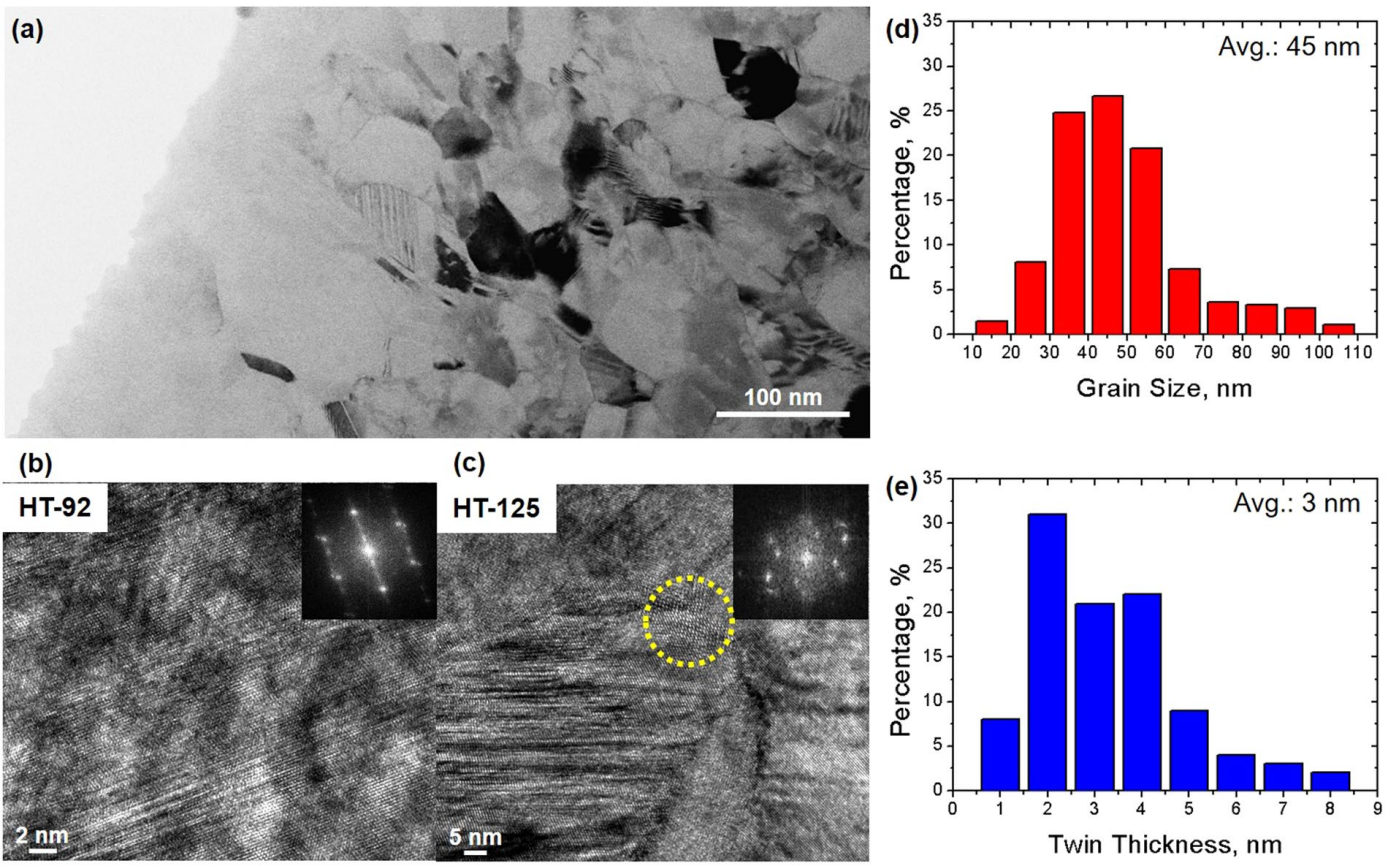
HT-HPT microstructures, showing the formation of deformation twins. **(a)** Low-magnification bright-field micrograph of the HT-HPT sample. **(b)** HRTEM images and corresponding selected area electron diffraction patterns with a [011]_fcc_ zone axis shown as an inset for HT-92 samples. The formation of nanometer-thick twin. **(c)** HRTEM micrograph for the HT-125 sample. Both nanometer-thick twins and nano-sized particle on grain boundary, highlighted by a yellow circle. The distributions of **(d)** grain size and **(e)** lamellar twin thicknesses, determined from HRTEM images, revealing an average grain size and twin thickness of 45 and 3 nm, respectively.

Figure 3(a–c) show the bright-field TEM images of two HT-92 and HT-125 samples, as well as the corresponding electron diffraction patterns obtained from the outlined area. The TEM image obtained from the HT-92 sample shows no solid phase transformation associated with the TRIP effect, but a regular or highly dense array of deformation twins. A remarkable observation on HT-125 is the occurrence of nano-sized particles <6 nm along the GBs of the fcc host matrix, as highlighted by a circle in Fig. 3(c). The formation of such nanoparticles is presumably due to solute diffusion along the GBs during the HT-HPT process, especially in *ε*_*s*_ = 125. Therefore, despite being strained over an extremely short period, the SPD process at ambient temperature may induce solute segregation along GBs in HT-125 as compared to the HT-92 sample. Grain size measurements in Fig. 3(d) shows a similar average size of approximately 45 nm for all HT-HPT samples, which is the same as the RT-HPT samples. Twin thickness measurements in Fig. 3(e) shows a similar distribution and a similar mean thickness of 3 nm for all HT-HPT samples. This observation suggests that strengthening in all HT-HPT samples do not exhibit a linear relationship to twin thickness. This observation on the deformation twin formation in the HT-HPT samples results from an increase in stacking fault energy (SFE). The SFE value is controlled by adjusting the chemical composition and temperature, and is a well-known criterion for the transition in deformation mechanisms from solid phase transformation to deformation twin. Above its critical value, an intermediate quantity of SFE leads to deformation twin formation, and thereby the twined material achieves plasticity through the so-called twinning-induced plasticity (TWIP) effect. Given that coherent twin boundaries can hinder dislocation gliding (dynamic Hall-Petch effect), they provide additional strength to the HT-HPT sample. Hence, we suggest that the TWIP effect in the HT-HPT material can offer greater strength at a given *ε*_*s*_ value than the TRIP effect (γ → ε) in the RT-HPT sample. A high SFE value in HT-HPT may originate from a high temperature compared to that of RT-HPT at the same *ε*_*s*_ values.

### APT analysis

**Figure 4:**
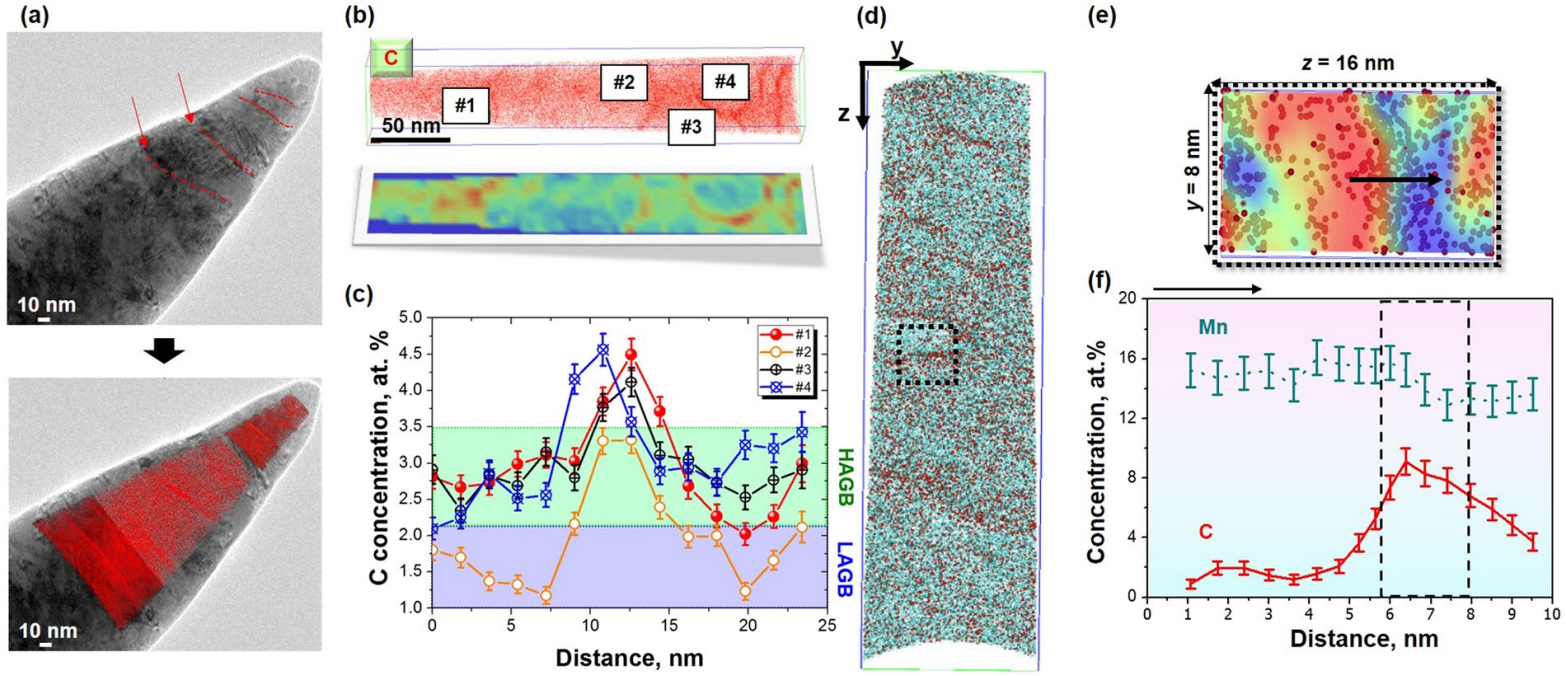
C segregation to grain boundaries in HT-125 sample. **(a)** Bright-field TEM image and correlative APT results of C (red) obtained from the same tip. Red arrows and dotted lines mark grain boundaries visible in both TEM micrograph and 3D map. **(b)** 3D C atom map and corresponding 2D contour map for visual correlation with the APT map of segregation to four different grain boundaries. **(c)** 1D compositional profiles across matrix-boundary interfaces highlighted in the atom map reveal that not all grain boundaries contain the same chemical composition of C. **(d)** The sectional Mn (cyan) and C (red) distributions (measured volume = 50 nm X 50 nm X 150 nm). **(e)** 2D contour map of Mn that is superimposed to C atoms near the line dislocation (the magnified area is marked as a solid line; measured area = 8 × 16 nm^2^; red: high-quantity of Mn, blue: low-quantity of Mn). **(f)** 1D compositional profiles show both Mn and C are segregated at the line dislocation.

To better understand the GB segregation, a complementary characterization through combining TEM and APT^35^ was performed on RT-125 and HT-125 (For RT-125, see the Supplementary Fig. 3). Figure 4 shows the summarization of C segregation to a grain boundary in HT-125 sample. This correlative TEM-APT results clearly show linear nature of structural defects and cross-correlated it with solute C redistribution in the three-dimensional reconstructions (Fig. 4(a)). As outlined by red arrows, the planar C-rich features in the TEM-APT volumes are identified as GBs. As represented in Fig. 4(b), solute C atoms that have strong tendency to segregate into all the GBs are approximately doubled as compared to those in the bulk matrix. Moreover, as represented in Fig. 4(c), not all GBs attract C atoms with an equal concentration to be detectable with APT, supporting the orientation-dependent nature of GBs in polycrystalline materials^18^. With the exception of C, no apparent elemental segregations can be observed in the APT results (See the Supplementary Fig. 4). Hence, it is plausible that the observed C accumulation at GBs in the HT-125 samples, referred to as GB-C, is the proof of the existence of C diffusion during the SPD process at relatively high temperature.

Besides the GBs, we demonstrate that dislocations can efficiently capture solutes in the HT-125 samples. Figure 4(d) shows the irregular distributions of C (red dots) and Mn (cyan dots) (arrows) in the 3D reconstructions that suggest linearly confined depletion or accumulation around dislocations, an effect referred to as the competing C−Mn migrating interactions at edge dislocations during the HT-HPT treatment as represented in Fig. 4(e)^36^. Both the 2D contour map of Mn, superimposed to the detected C atoms, and the 1D concentration profile reveal the C−Mn interactions around the dislocations (Fig. 4(f)): C depletion at high densities of Mn (red color region), and vice versa, C accumulation at low densities of Mn (blue color region). As a result, the solute atoms induce short-range ordering in the γ-austenite matrix and interact with dislocations as follows^37^: (i) when the edge dislocation exists in an fcc structure, the tensile pressure occurs at the vacant region while the compressive pressure induces at the extra-half plane^38^. (ii) The segregated solutes reduce the dislocation mobility during plastic deformation, hampering the dislocation movement.

The driving force for GB-C in the HT-HPT samples, originating from the substantial excess of temperature and mobile dislocations in nano-sized grains, becomes sufficiently large for GB-C to occur easily, even at an ambient temperature of 573 K. Assuming that this phenomenon is sufficiently pronounced to represent the well-known pipe diffusion theory, the calculated diffusion mean free path of C and Mn at 573 K are 3.65 μm and 53 nm, respectively^39^. Taking into account the average grain size, the calculated diffusion mean free path suggests that GB segregation is more prevalent in C atoms than in Mn atoms, whereas the dislocation segregation is induced by both C and Mn atoms. These segregated defects suppress the dislocation emission, and the additional strengthening leads to strength enhancement in the HT-125 sample.

## Discussion

**Figure 5:**
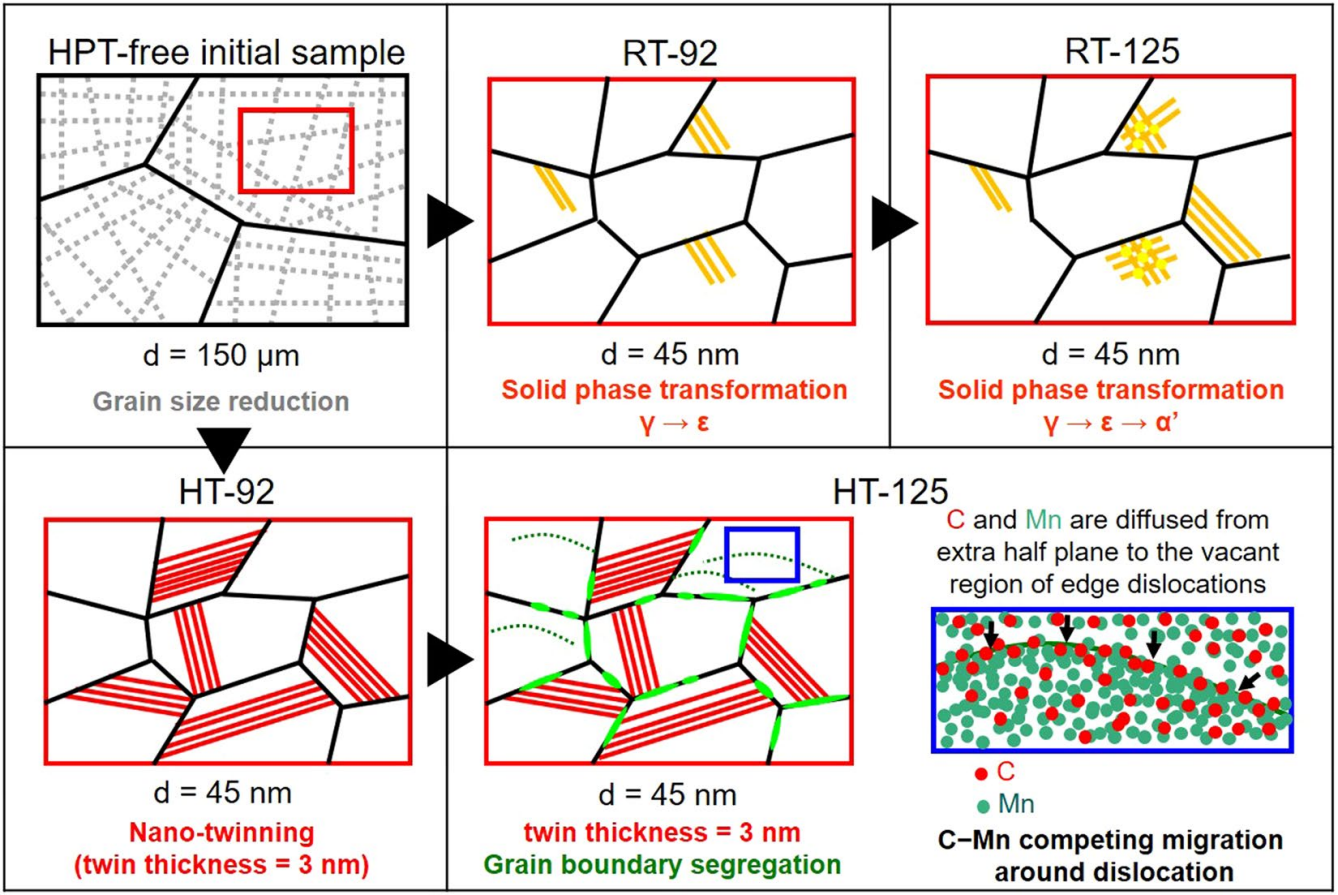
Schematic diagram showing dominating strengthening mechanism evolution steps of HT- and RT-HPT samples. For *ε*_*s*_ < 92 (Stage I), grain refinement occurs in all HPT-treated samples and grain size remains unchanged at a given HPT temperature. (ii) For *ε*_*s*_ = 92 (Stage II), the RT-HPT sample achieves plasticity by means of solid phase transformation, while nanometer-thick twins occur in the HT-HPT sample. (iii) For *ε*_*s*_ = 125 (Stage III), multiple phases coexist by means of two-step paths of solid phase transformation for RT-HPT sample, while nano-twins, nano-grains, and solute segregation occur for the HT-HPT sample.

The microstructural characterization of two different NC materials (RT-HPT and HT-HPT) from the micrometer to atomic scale provides an accessible pathway for understanding their multi-strengthening mechanisms. As represented in Fig. 5, the stepwise multi-strengthening mechanisms of combining temperature and high shear straining during the HPT treatment are as follows, (i) for *ε*_*s*_ < 92 (Stage I), the strength of all HPT-treated samples increases with increasing *ε*_*s*_ values. In this stage, the Hall-Petch effect via grain size refinement from 150 μm to 45 nm is the dominating strengthening mechanism. (ii) For *ε*_*s*_ = 92 (Stage II), the hardness and strength of the HT-HPT samples are higher than those of the RT-HPT materials. The strengthening mechanisms in each HPT sample is differentiated by the HPT-applied temperature: in the RT-92 sample, solid phase transformation of γ → ε occurs by means of colliding *a*/6 < 112 > partial dislocations in the fcc host matrix^40^; in the HT-92 material, high densities of nano-twins with an average thickness of 3 nm are introduced into nano-sized grains, however, no solid phase transformation occurs. Because of the differentiated strengthening mechanisms, the strength difference between the HT- and RT-HPT samples is evolved from this stage. (iii) In the highly-strained state (92 ≤ *ε*_*s*_ ≤ 125; Stage III), the greatest hardness and strength were obtained in the HT-125 sample, whereas there were no obvious changes in the properties of the RT-125 sample. Because the thickness of deformation twins in the HT-125 sample remains unchanged compared to that of the HT-92 counterpart, the contribution of twin boundaries to strength is not efficient. Instead, both the grain boundary segregation via C migration and the dislocation pinning via C−Mn competing migration behavior hinder the predominant dislocation gliding during plastic deformation. This continuous solute redistribution at crystal defects during the HT-HPT treatment provides extra strength to the HT-125 sample.

The experimental data obtained convincingly testify that severe straining of the investigated steel by HPT results in its remarkable microstructure transformation. These changes are related to tremendous grain refinement down to nanometer range as well as to phase transformations. Let us attempt to analyze the observed radical increase in strength in terms of contribution of different hardening mechanisms in the UFG materials with complicated microstructure. Traditionally, the additive theory is used to account for every obstacle capable to be a barrier for the dislocation motion so that the total yield stress is calculated as a simple sum of any corresponding hardening contribution. Several attempts were made to estimate the yields stress in a different^41^, however, the additive theory is commonly used in literature to evaluate hardening induced by different microstructure components.

Thus, we intend to separate and discuss the impact of different nanostructural features to the strength and to analyze if the revealed features are enough to explain its huge increase. According to the present result, the strengthening mechanisms of HT-125 sample can be divided into (i) grain size effect, (ii) dislocation effect, (iii) solid solution effect, (iv) twin boundary effect, and (v) segregation effect. Each strengthening mechanism contributions can be estimated by calculating the proportion of strength in the HT-125 sample.

The grain size dependent strengthening (*σ*_*HP*_) can be estimated by the Hall-Petch equation:

1 $${\sigma }_{HP}={K}_{HP}{d}^{-0.5},$$

where *K*_*HP*_ is the Hall-Petch constant and *d* is the average grain size. The *K*_*HP*_ of high manganese steel is 0.357 MPa m^0.5^ and *d* is measured to 45 nm^42^. Based on these variables, the strength from the grain size effect is equivalent to 1682.9 MPa.

The dislocation density-based strengthening in the metallic material describes the strengthening arising out of the intersection of dislocations during plastic deformation. The strength from the dislocation density (*σ*_*D*_) is derived as follows^43^:

2 $${\sigma }_{D}=M\alpha Gb{\rho }^{0.5},$$

where *M* is the Taylor factor, *α* is a constant, *G* is the shear modulus, *b* is the burgers vector, and *ρ* is the dislocation density. The previous reports revealed that the *M* of FCC material is equivalent to 3, *α* is 0.3, the *G* of present materials is 65000 MPa, and the *b* is 0.25 nm^44^. The *ρ* of HT-HPT sample was measured by the convolutional multiple whole profile method^45^, and estimated to be 1.57 $\times$ 10^15^ m^−2^. Based on these variables, the *σ*_*D*_ is equivalent to 579.5 MPa.

The solid solution strengthening (*σ*_*S*_) by the substitutional solute can be estimated as follows^46^:

3 $${\sigma }_{S}=AG{\varepsilon }^{4/3}{C}^{2/3},$$

where *A* is the dimensionless parameter, *G* is the shear modulus, *ε* is the lattice strain due to the size difference between solute and matrix atoms, *C* is the concentration of solute atoms. The *A* is the order of 0.1, *G* is 65000 MPa, *C* is 0.15, and the atomic size difference between Mn (atom radii = 161 pm) and Fe (atom radii = 156 pm) is 3%. Based on these variables, the *σ*_*S*_ of present sample is 17.1 MPa.

The twin boundaries act as a grain boundary, and its strengthening contribution can be treated as Hall-Petch type strengthening. The twin boundary strengthening (*σ*_*TW*_) can be estimated as follows^47^:

4 $${\sigma }_{TW}=F{K}_{TW}{l}^{-0.5},$$

where *F* is the volume fraction of twins, *K*_*TW*_ is the Hall-Petch coefficient of twin boundaries, and *l* is the twin spacing. The previous report revealed that the *K*_*TW*_ ≤ *K*_*HP*_ relation occurs in TWIP steel^48^. This means that the twinning stress is originated from the slip resistance associated with grain boundaries. In this research, the *K*_*TW*_ is assumed to same as the *K*_*HP*_, 0.357 MPa m^0.5^^49^. *F* can be estimated by the stereological analysis of Fullman as follows^50^:

5 $$\frac{1}{l}=\frac{1}{2e}\frac{F}{1-F},$$

where *e* is the twin thickness. In the present sample, we observed that the *l* is 43 nm and the *e* is 3 nm. Based on the observation results, we can estimate that the *F* is equivalent to 0.122. Therefore, the *σ*_*TW*_ is 210 MPa.

The GB segregation acts as an additional strengthening mechanism in two ways: (i) The segregated solute atoms improve the grain-to-grain coherency^15^, and (ii) solute clusters at the grain boundary can act as an obstacle for dislocation gliding^14^. From these reasons, GB segregation interrupts dislocation near the grain boundary and increases *K*_*HP*_ of materials. In the present APT analysis, we found that the C contents at the GB of the nanosteel is 4 at% (equivalent to 0.9 wt%) while the γ-austenite matrix contains 2.6 at% C. Since the present model steel 15Mn-0.6C-1.5Al contains 2.68 at% C, the present APT result is reliable. In the conventional high Mn steel, C segregation was not observed at the GB^51^. Such result allows making an assumption that the C contents at the GB of conventional high Mn steel are similar to that in the matrix (2.68 at%). According to the previous report, the *K*_*HP*_ of austenitic steels has a linear relation with its C wt% contents^52^. From this result, we can quantify that the *K*_*HP*_ of nanosteel (0.9 wt% C @ GBs) is 1.2 times of conventional steel (0.6 wt% C @ GBs). If we apply it to the present Hall-Petch calculation, GB segregation provides 320 MPa to the nanosteel.

Based on the calculated strengthening mechanism contributions, we know that the grain refinement, dislocation density, solid solution, twin boundary strengthening, and GB segregation contribute to the high strength in the HT-125 sample. The summation of strength from each strengthening mechanism is equivalent to 2810 MPa, which is larger than the experimental value (2.6 GPa). Such an overestimated result can be explained in two ways: (i) The *K*_*TW*_ is smaller than *K*_*HP*_^46^. This means that the calculated *σ*_*TW*_ is overestimated. (ii) The measured C contents at the GBs contains inescapable errors (~0.3 at%) in APT. Therefore, the quantified *K*_*HP*_ of the nanosteel also have some errors. Although these two variables produce some errors in the calculation results, the estimated strength shows a good correlation with the experimental results. This means that all of the strengthening mechanisms contribute to the strength of nanosteel. Based on the calculated strengthening mechanism contributions, we conclude that the grain size refinement, dislocation density, solid solution, twin boundary strengthening, and GB segregation contribute to the high strength in the HT-125 sample.

## Conclusion

In conclusion, the ultra-strength of the investigated nanosteel has been obtained by adopting stepwise multiple strengthening activities via HPT processes at different temperatures. Based on our analyses, the room temperature HPT-treated material that contains refined domain structures with high-density crystal defects exhibits the 2.3 GPa strength in terms of the deformation-stimulated solid phase transformation. In addition, the strength of the nanograined material can be raised further by employing elevated temperature HPT (by 2.6 GPa). This strength increase can be attributed to a combination of multiple strengthening mechanisms, such as the formation of nanoscale twins, segregation of C to grain boundaries, and the atomic rearrangement of C and Mn around the dislocation. The calculated strengthening mechanism contributions represent that all of the strengthening mechanisms contribute to the strength of nanosteel. Thus, we have provided insight into developing a wide range of advanced high-strength nanotwinned and nanostructured materials.

## Methods

### Sample preparation

The model steel is an Fe−15 Mn−0.6 C−1.5 Al by weight percent. The alloy was solution-treated at 1200 °C for 2 h, and hot-rolled in the range of 1100−900 °C from 40 to 2.5 mm thickness. To simulate coiling, the sample was heated at 450 °C for 1 h and furnace-cooled. To adjust the grain size of model alloy, an annealing process was conducted at 1200 °C for 2 h with a cooling rate of 25 °C/s (Detailed mechanical and microstructural properties of the model alloy are represented in Supplementary Fig. 5). The HPT process was performed at 298 K (RT-HPT) and 573 K (HT-HPT) by imposing a pressure of 6 GPa over 10 revolutions (0.2 rpm) to the disk-shaped specimens of 10.0 mm diameter and 2.5 mm thickness.

### Mechanical testing

The local mechanical properties of the HPT samples were measured using a Vickers microhardness tester (FM-700, Future Tech., Japan). The dwell load and time were 300 gf and 10 s, respectively. Tensile specimens with a gage length of 0.5 mm were cut from a *D*_*f-c*_ = 2.5 and 3.5 mm for tensile testing. The tensile tests of all specimens in both the RT- and HT-HPT state were conducted using a universal testing machine (Instron 1361, Instron Corp., Canton, USA) at a quasi-static strain rate of 10^−3^ s^−1^ at 298 K.

### TEM observation

For TEM observations, samples were prepared using a dual-beam focused ion beam (FIB, FEI Helios Nano-Lab^TM^). Bright-field low- or high-magnified images and high-resolution TEM (HRTEM) images were obtained with a JEOL-2200FS analytical TEM equipped with an aberration corrector that was operated at an acceleration voltage of 200 kV. To obtain the diffraction patterns of the HPT treated samples, we employed fast Fourier transformation (FFT) to filter the noise from the lattice image, and then performed inverse (IFFT) characterization of the targeted area.

### Local electrode APT analysis

Samples for APT analyses were prepared by electropolishing, followed by treatment with a FIB (FEI, Helios Nano-Lab 600). APT measurements were taken using a local electrode atom probe (LEAP 4000X HR, CAMECATM) in the voltage-pulsing mode. The experimental parameters were set to maintain a 0.2% detection rate, 20% pulse fraction, and 200-kHz pulse repetition. All measurements were performed at 40 K at < 10^−7^ Pa pressure. A minimum of two successful measurements were performed. The APT data sets were mapped in 3D using IVAS software (version 3.6.10) supplied by Cameca Instruments. Reconstruction was calibrated by determining the APT sample geometric parameters, such as tip radius and shank angle. Statistical errors for measured atom counts were calculated as σ = (C_i_ × (1 − C_i_)/N)^−1/2^, where C_i_ corresponds to the measured atomic concentration fraction of the individual element i, and N is the total number of atoms collected in the bin.

## Electronic supplementary material

Supplementary Materials

## References

[1] Elias CN, Lima JHC, Valiev R, Meyers MA (2008). Biomedical applications of titanium and its alloys. JOM.

[2] Yanagida A, Joko K, Azushima A (2008). Formability of steels subjected to cold ECAE process. J. Mater. Process. Technol..

[3] Nieh TG, Wadsworth J (1991). Hall-Petch relation in nanocrystalline solids. Scr. Metall. Mater..

[4] Weertman JR (1999). Structure and mechanical behavior of bulk nanocrystalline materials. MRS Bull..

[5] Karavaeva MV (2015). Superior strength of carbon steel with an ultrafine-grained microstructure and its enhanced thermal stability. J. Mater. Sci..

[6] Qin S (2016). Ultrahigh ductility, high-carbon martensitic steel. Metall. Mater. Trans. A.

[7] Kuzmina M, Herbig M, Ponge D, Sandlöbes S, Raabe D (2015). Linear complexions: Confined chemical and structural states at dislocations. Science.

[8] Khalajhedayati A, Pan Z, Rupert TJ (2016). Manipulating the interfacial structure of nanomaterials to achieve a unique combination of strength and ductility. Nat. Commun..

[9] Seol JB (2017). Core-shell nanoparticle arrays double the strength of steel. Sci. Rep..

[10] Lu L, Shen Y, Chen X, Qian L, Lu K (2004). Ultrahigh strength and high electrical conductivity in copper. Science.

[11] Wang Y, Chen M, Zhou F, Ma E (2002). High tensile ductility in a nanostructured metal. Nature.

[12] Kou H, Lu J, Li Y (2016). High-Strength and High-Ductility Nanostructured and Amorphous Metallic Materials. Adv. Mater..

[13] Valiev RZ, Enikeev NA, Murashkin MY, Kazykhanov VU, Sauvage X (2010). On the origin of the extremely high strength of ultrafine-grained Al alloys produced by severe plastic deformation. Scr. Mater..

[14] Abramova MM (2014). Grain boundary segregation induced strengthening of an ultrafine-grained austenitic stainless steel. Mater. Lett..

[15] Raabe D (2014). Grain boundary segregation engineering in metallic alloys: A pathway to the design of interfaces. Curr. Opin. Solid State Mater. Sci..

[16] Djaziri S (2016). Deformation-Induced Martensite: A New Paradigm for Exceptional Steels. Adv. Mater..

[17] Kang JH, Duan S, Kim SJ, Bleck W (2016). Grain Boundary Strengthening in High Mn Austenitic Steels. Metall. Mater. Trans. A.

[18] Herbig M (2014). Atomic-scale quantification of grain boundary segregation in nanocrystalline material. Phys. Rev. Lett..

[19] Zhilyaev A, Langdon TG (2008). Using high-pressure torsion for metal processing: Fundamentals and applications. Prog. Mater. Sci..

[20] Oberdorfer B (2014). Grain boundary excess volume and defect annealing of copper after high-pressure torsion. Acta Mater..

[21] Um HY (2014). Hollow cone high-pressure torsion: Microstructure and tensile strength by unique severe plastic deformation. Scr. Mater..

[22] Ivanseko Y (2016). High pressure torsion extrusion as a new severe plastic deformation process. Mater. Sci. Eng. A.

[23] Takizawa Y (2016). Scaling up of high-pressure sliding (HPS) for grain refinement and superplasticity. Metall. Mater. Trans. A.

[24] Lee HH, Yoon JI, Kim HS (2018). Single-roll angular-rolling: A new continuous severe plastic deformation process for metal sheets. Scr. Mater..

[25] An XH (2016). Microstructural evolution and phase transformation in twinning-induced plasticity steel induced by high-pressure torsion. Acta Mater..

[26] Gault, B. *et al*. Atom Probe Microscopy. Springer Science + Business Media. (2012).

[27] Zhilyaev AP (2003). Experimental parameters influencing grain refinement and microstructural evolution during high-pressure torsion. Acta Mater..

[28] Lee DJ, Kim HS (2014). Finite element analysis for the geometry effect on strain inhomogeneity during high-pressure torsion. J. Mater. Sci..

[29] Lü Y, Hutchinson B, Molodov DA, Gottstein G (2010). Effect of deformation and annealing on the formation and reversion of ε-martensite in an Fe–Mn–C alloy. Acta Mater..

[30] Fujita H, Ueda S (1972). Stacking faults and F.C.C. (γ) → H.C.P. (ε) transformation in 18/8-type stainless steel. Acta Metall..

[31] Remy L, Pineau A (1977). Twinning and strain-induce F.C.C. → H.C.P. transformation in the Fe-Mn-Cr-C system. Mater. Sci. Eng..

[32] Grässel O, Krüger L, Frommeyer G, Meyer LW (2000). High strength Fe–Mn–(Al, Si) TRIP/TWIP steels development-properties-application. Int. J. Plast..

[33] Adachi N, Wu N, Todaka Y, Sato H, Ueji R (2016). Phase transformation in Fe-Mn-C alloys by severe plastic deformation under high pressure. Mater. Lett..

[34] Wu XL, Yang MX, Yuan FP, Chen L, Zhu YT (2016). Combining gradient structure and TRIP effect to produce austenite stainless steel with high strength and ductility. Acta Mater..

[35] Herbig M, Choi P, Raabe D (2015). Combining structural and chemical information at the nanometer scale by correlative transmission electron microscopy and atom probe tomography. Ultramicroscopy.

[36] Lee SJ, Kim J, Kane SN, De Cooman BC (2011). On the origin of dynamic strain aging in twinning-induced plasticity steels. Acta Mater..

[37] Seol JB, Kim JG, Na SH, Park CG, Kim HS (2017). Deformation rate controls atomic-scale dynamic strain aging and phase transformation in high Mn TRIP steels. Acta Mater..

[38] Hu SY, Chen LQ (2001). Solute segregation and coherent nucleation and growth near a dislocation-a phase-field model integrating defect and phase microstructures. Acta Mater..

[39] Seo SW, Bhadeshia H, Suh DW (2015). Pearlite growth rate in Fe–C and Fe–Mn–C steels. Mater. Sci. Technol..

[40] Brooks JW, Loretto MH, Smallman RE (1979). Direct observations of martensite nuclei in stainless steel. Acta Metall..

[41] Morris, D. G. Strengthening mechanisms in nanocrystalline metals: in Nanostructured Metals and Alloys: Processing, Microstructure, Mechanical Properties and Application. Woodhead Publishing Co. 299–328 (2011).

[42] Sevillano JG (2009). An alternative model for the strain hardening of FCC alloys that twin, validated for twinning-induced plasticity steel. Scr. Mater..

[43] Estrin Y, Mecking H (1984). A unified phenomenological description of work hardening and creep based on one-parameter models. Acta Metall..

[44] Bouaziz O, Allain S, Scott S (2008). Effect of grain and twin boundaries on the hardening mechanisms of twinning-induced plasticity steels. Scr. Mater..

[45] Ungar T, Tichy G (1999). The effect of dislocation contrast on X-ray line profiles in untextured polycrystals. Phys. Status Solidi A.

[46] Senkov ON, Scott JM, Senkova SV, Miracle DB, Woodward CF (2011). Microstructure and room temperature properties of a high-entropy TaNbHfZrTi alloy. J. Alloy. Compd..

[47] Ganji RS, Karthik PS, Rao KBS, Rajulapati KV (2017). Strenthening mechanisms in equiatomic ultrafine grained AlCoCrCuFeNi high-entropy alloy studied by micro- and nanoindentation methods. Acta Mater..

[48] Gutierrez-Urrutia I, Zaefferer S, Raabe D (2010). The effect of grain size and grain orientation on deformation twinning in a Fe-22 wt.% Mn-0.6 wt.% C TWIP steel. Mater. Sci. Eng. A.

[49] Des Las Cuevas F (2010). Hall-Petch relationship of a TWIP steel. Key Eng. Mater..

[50] Fullman RL (1953). Measurement of particle sizes in opaque bodies. Trans. AIME..

[51] Jin J-E, Lee Y-K (2012). Effects of Al on microstructure and tensile properties of C-bearing high Mn TWIP steel. Acta Mater..

[52] Kang J-H, Duan S, Kim S-J, Bleck W (2016). Grain boundary strengthening in high Mn austenitic steels. Metall. Mater. Trans. A.

