# Supplementary material for: Superior Strength and Multiple Strengthening Mechanisms in Nanocrystalline TWIP Steel
Source: Sci Rep. 2018 Jul 25;8:11200. doi: 10.1038/s41598-018-29632-y (PMC6060181; doi:10.1038/s41598-018-29632-y)
Supplement: Supplementary file 1 — Supplementary Materials [file 41598_2018_29632_MOESM1_ESM.docx]

**Supplementary Material**

**Superior Strength and Multiple Strengthening Mechanisms in Nanocrystalline TWIP Steel**

Jung Gi Kim^a,†^, Nariman A. Enikeev^b,c,†^, Jae Bok Seol^d^, Marina M. Abramova^b^, Marina V. Karavaeva^b^, Ruslan Z. Valiev^b,c^, Chan Gyung Park^a,d^ and Hyoung Seop Kim^a,e,*^

*^a^Department of Materials Science and Engineering, Pohang University of Science and Technology (POSTECH), Pohang 37673, South Korea*

*^b^Institute of Physics of Advanced Materials, Ufa State Aviation Technical University, Ufa, 450000, Russia*

*^c^Saint Petersburg University, St. Petersburg, 198504, Russia*

*^d^National Institute of Nanomaterials Technology (NINT), POSTECH, Pohang 37673, South Korea*

*^e^Center for High Entropy Alloys, POSTECH, Pohang 37673, South Korea*

^†^Authors contributed equally to this work.

*Corresponding Author: H.S. Kim, hskim@postech.ac.kr

**Contents**

Supplementary Figure 1

Supplementary Figure 2

Supplementary Figure 3

Supplementary Figure 4

Supplementary Figure 5

Supplementary Notes

Supplementary References

**Supplementary Figure 1**

**Figure S1.** Strain hardening rate-strain (*dσ/dε – ε*) curves and true stress-strain (*σ – ε*) curves of HT-92 and RT-92 HPT samples. Based on the Considere criterion, intersection between *dσ/dε* and *σ* can be treated as a uniform elongation (*ε*_u_). The *ε*_u_ of HT- and RT-HPT samples are 0.023-0.033.

**Supplementary Figure 2**


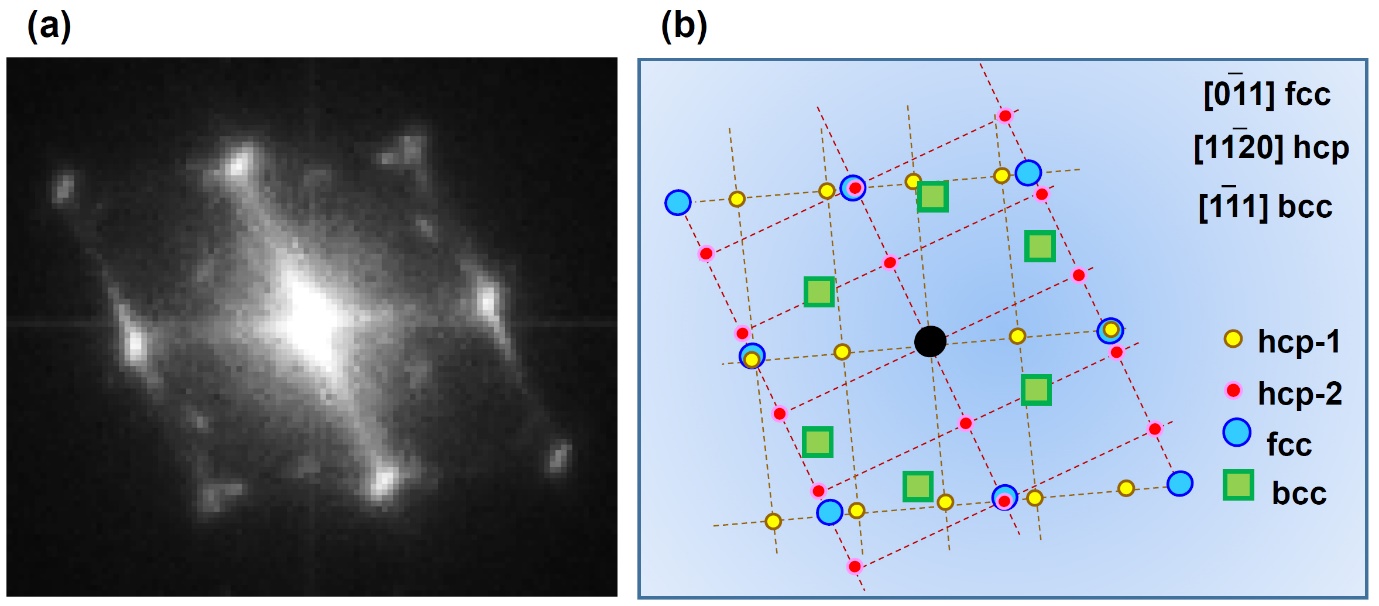


**Figure S2.** Fourier transformation diffraction pattern at *ε_s_* = 125 in the RT-HPT sample. (a) The expanded diffraction pattern image in Fig. 2(d). (b) The summary of the diffraction patterns. The diffraction pattern shows that the RT-HPT sample has (0$\bar{\boldsymbol{1}}$1) γ-austenite + (11$\bar{\boldsymbol{2}}$0) ε-martensite + (1$\bar{\boldsymbol{1}}$1) α’-martensite phases and all the phases have a correlative relation at the [011]_fcc_ zone axis.

**Supplementary Figure 3**


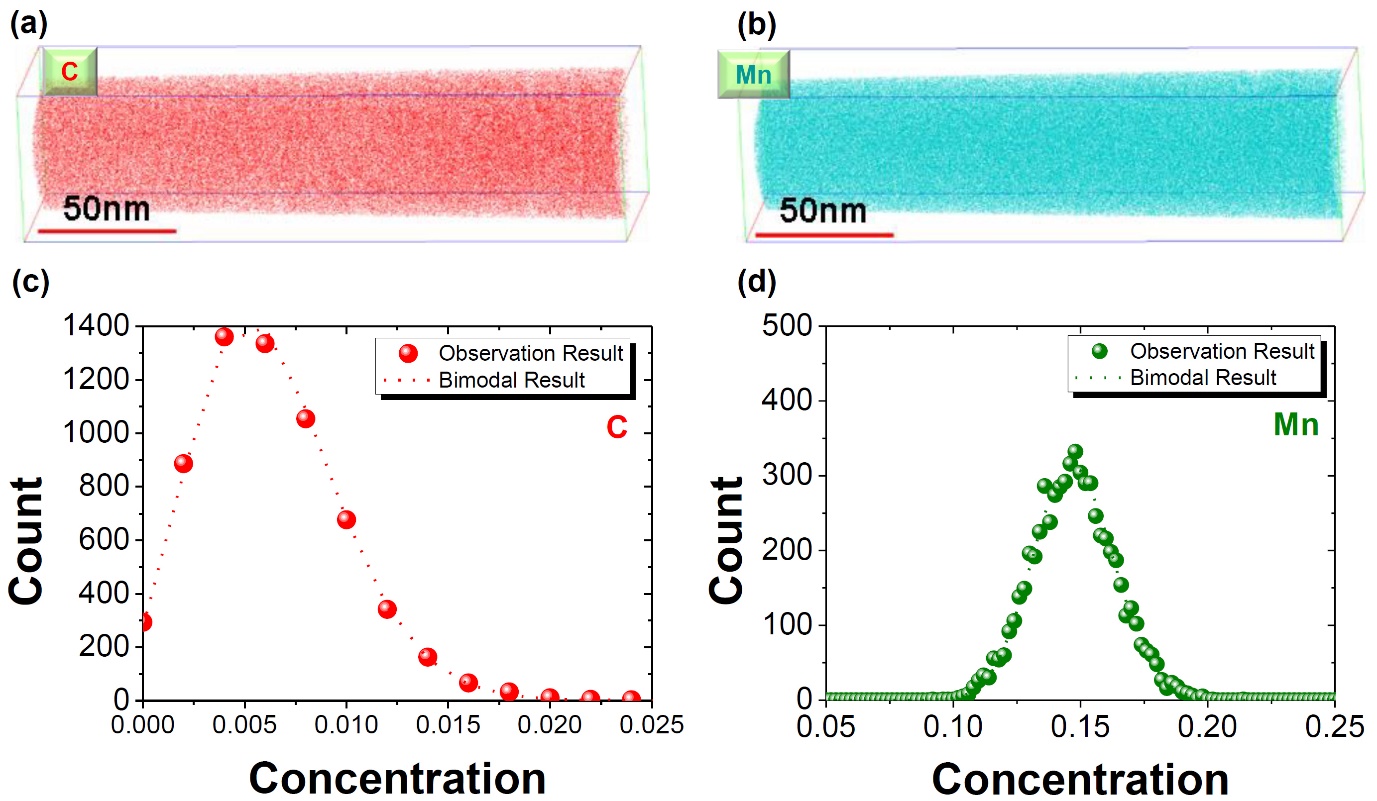


**Figure S3.** Atom probe tomography results of the RT-HPT sample. (a) C / (b) Mn atomic structures at the *ε_s_* =125 position in the RT-HPT sample (Measured volume = 50 nm X 50 nm X 90 nm). No significant C and Mn segregations were observed in the RT-HPT sample. (c) C / (d) Mn bimodal distributions of the RT-HPT sample show the disordering is not induced and atoms are homogeneously distributed.

**Supplementary Figure 4**

**
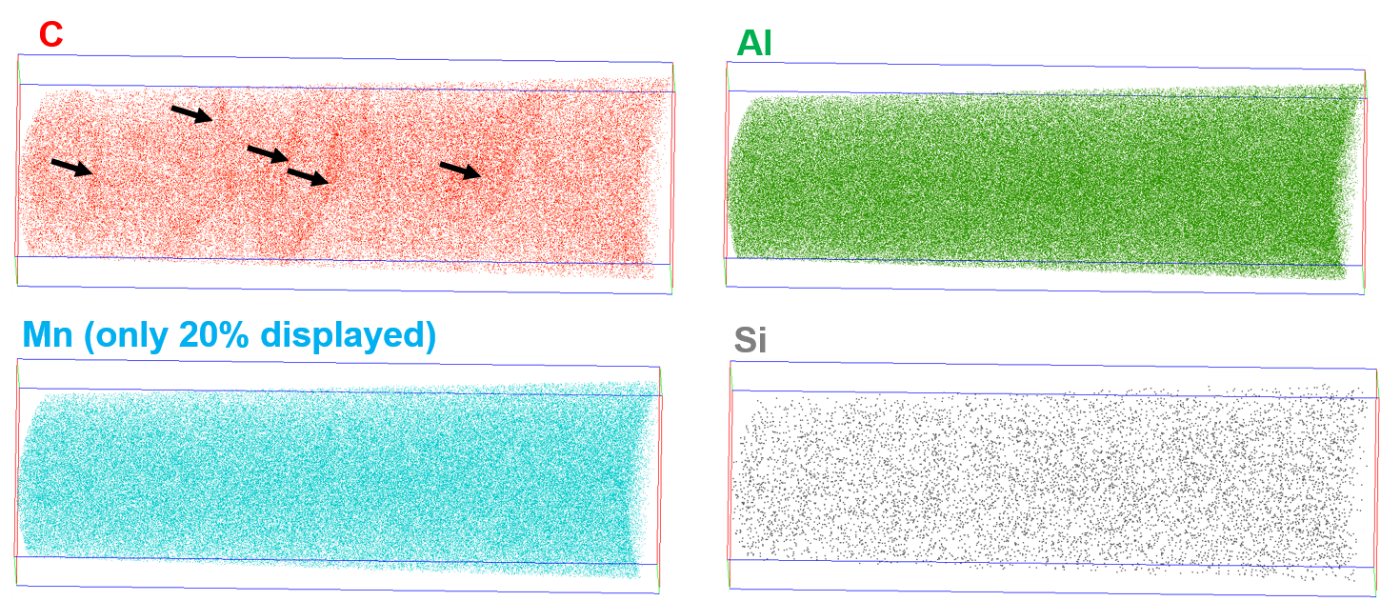
**

**Figure S4.** Atom probe tomography results of the HT-125 sample. The C atoms are segregated in the grain boundaries (marked as arrows) while the other atoms (Al, Mn, and Si) are homogeneously distributed. This result shows that the grain boundary segregation in HT-125 sample is originated from the C atom migration.

**Supplementary Figure 5**


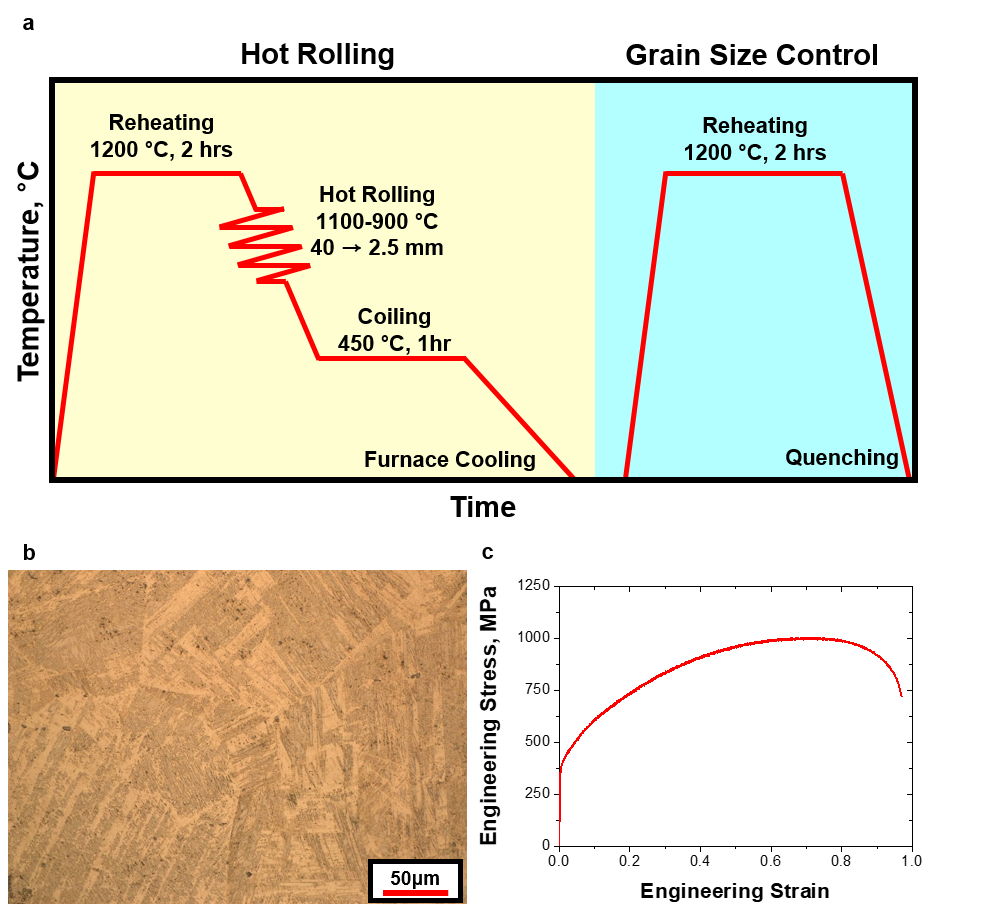


**Figure S5.** Summary of the processing and properties of the initial sample. (a) The processing map for preparing the initial sample. To adjust the initial grain size, the post-annealing process is included. (b) The morphology by the optical microscope. The average grain size of the initial sample is 150 μm. (c) The stress-strain curve of the initial sample.

**Supplementary Notes**

Additional explanation for Figure S3

Figure S3 shows that no solute-enriched regions were visible in the APT results of the RT-125 samples, which were determined by bimodal distribution analysis. This means that all solute elements remain homogeneously in solid solution in the RT-125 sample (i.e., segregation-free state)^1^. Hence, it is plausible that the observed C accumulation at GBs in the HT-125 samples, referred to as GB-C, is proof of the existence of C diffusion during the SPD process at a relatively higher temperature compared to that of the RT-125 samples.

**Supplementary Reference**

1. Stephenson, L.T. *et al*. New techniques for the analysis of fine-scaled clustering phenomena within atom probe tomography (APT) data. *Microsc. Microanal.* **13**, 448-463 (2017).
